# From Confidence to Coverage: A Multi-Stakeholder Perspective on Vaccination in Central and Eastern Europe

**DOI:** 10.3390/vaccines14080678

**Published:** 2026-08-06

**Authors:** Teodor Cristian Blidaru, David Sinclair, Petr Smejkal, Yasmin Maor, Ernest Kuchar, Catherine Weil-Olivier, Mariano Votta, Luminița Vâlcea, Valeriu Gheorghiță

**Affiliations:** 1 Faculty of Medicine, “Carol Davila” University of Medicine and Pharmacy, Dionisie Lupu 37, 020021 Bucharest, Romania; 2 International Longevity Centre UK (ILC-UK), The Foundry, 17 Oval Way, London SE11 5RR, UK; 3 Department of Hygiene and Epidemiology, Institute for Clinical and Experimental Medicine (IKEM), Vídeňská 1958/9, 140 21 Prague, Czech Republic; 4 Infectious Disease Unit, E. Wolfson Medical Center, HaLochamim St. 62, Holon 5822012, Israel; 5 Gray Faculty of Medical and Health Sciences, Tel Aviv University, Ramat Aviv, Tel Aviv 6997801, Israel; 6 Department of Paediatrics with Clinical Assessment Unit, Medical University of Warsaw, Żwirki i Wigury 63A, 02-091 Warsaw, Poland; ernest.kuchar@gmail.com; 7 Université Paris Cité, 85 Boulevard Saint-Germain, 75006 Paris, France; 8 Active Citizenship Network–Cittadinanzattiva, Via Imera 2, 00183 Rome, Italy; m.votta@cittadinanzattiva.it; 9 COPAC—Coalition of Organisations of Patients with Chronic Diseases in Romania, Str. Țepeș Vodă 22, 021525 Bucharest, Romania; 10 “Agrippa Ionescu” Clinical Emergency Hospital, Str. Arh. Ion Mincu 7, 011356 Bucharest, Romania

**Keywords:** vaccine coverage, vaccine uptake, vaccine hesitancy, vaccine confidence, immunisation strategies, Central and Eastern Europe, health communication, primary care, health equity, health systems, digital health, health policy

## Abstract

Central and Eastern Europe (CEE) carries some of the widest immunisation gaps in Europe, and improving coverage there is often treated as a problem of access infrastructure. On 14 May 2026, a multi-stakeholder meeting hosted at the Romanian Parliament brought together around twenty-five speakers, among some fifty participants in all, from Romania, Poland, the Czech Republic, and Bulgaria, together with international contributors, to agree which barriers to vaccine confidence matter most in the region, which interventions are deliverable within 12 to 24 months, and how responsibility for them should be divided among stakeholders. This Perspective synthesises that discussion as a set of strategies for coverage. Meeting-derived priorities are set against published coverage and confidence data for the region and against the published intervention literature. We argue that the central problem across the region is eroded trust rather than missing information, because misinformation succeeds mainly where trust has already broken down, a process deepened by a historically conditioned, post-communist distrust of public institutions. The two recommendations carried most strongly, as both high in expected impact and achievable within 12 to 24 months, were continuing professional education for primary care on vaccine communication and the resourcing of locally trusted community figures, including health mediators working with marginalised populations. We then show how system-design reforms already demonstrated in the region, in particular pharmacy-based vaccination, direct reimbursement, and targeted outreach to under-vaccinated and marginalised groups, turn restored confidence into measurable coverage while narrowing inequities. We close with a 12-to-24-month agenda for CEE, organised around a clear multi-stakeholder division of labour. These are stakeholder-derived priorities that now require implementation and evaluation.

## 1. Introduction

Vaccines remain among the most cost-effective tools in public health, yet immunity gaps persist and, in several countries, are widening. In 2024 and 2025, the World Health Organization and UNICEF warned that immunisation progress was under renewed threat, leaving millions of children, adolescents, and adults exposed to vaccine-preventable disease [1]. Central and Eastern Europe (CEE) sits at the sharper end of this trend. Childhood coverage has been declining in parts of the region since the late 2000s, adult immunisation programmes remain scarce, and pandemic-era uptake lagged well behind the European average. The measles indicator makes the gradient concrete. In Romania, coverage with the first dose of measles-containing vaccine fell from 96–97% in 2005–2008 to 90.0% in 2019 and 83.4% in 2022, while second-dose coverage fell from the same baseline to 75.8% and then 71.4%, reaching roughly 62% by 2023, against a European average of about 89% and the 95% threshold required to interrupt transmission [2]. Sub-national variation is wider still: 73% of Romania’s 42 counties were below the 75% first-dose coverage at 12 months in 2022, compared with 38% in 2021 [2]. Poland shows the same direction through a different indicator, with recorded refusals of mandatory childhood vaccination rising from 3437 in 2010 to 48,600 in 2019 and 87,300 in 2023 [3]. The consequences are epidemiological rather than hypothetical: Romania reported 7243 measles cases and eight deaths between January 2023 and March 2024, and accounted for the large majority of measles cases and of paediatric measles deaths recorded in the EU/EEA over that period [2]. Measles is used here as the sentinel indicator because it requires the highest population immunity to interrupt transmission and is the most sensitive marker of programme performance, but the decline is not confined to it: in Romania, third-dose coverage with the hexavalent diphtheria–tetanus–pertussis–hepatitis B–Hib–poliovirus vaccine that forms the backbone of the infant schedule fell from 90% in 2019 to 85% in 2022 and 78% in 2023, and third-dose poliovirus coverage followed the same path, each dropping below the 90–95% targets [1]. Beneath these figures lies a decline in confidence itself: trust in the health system is far lower than in the rest of the EU, with low trust reported by close to half of citizens in parts of Eastern Europe, against around one fifth across the EU as a whole [4]. The same east–west gradient appears in attitudes as well as in coverage: in the 2019 Special Eurobarometer on vaccination, agreement with statements on vaccine safety and importance was consistently lower in Romania and Bulgaria than in western and northern Member States [5], and a structured review of published evidence from seven Central and Eastern European countries found confidence across the region to be persistently below the western European average [6].

The common framing attributes these gaps mainly to access, that is, to distance, supply and cost. The evidence assembled here suggests that this framing is incomplete. The recurrent theme across countries and professional roles was instead confidence, meaning the willingness of individuals and communities to accept a recommended vaccine. Confidence in this sense is relational rather than informational, because it depends on three distinct relationships: whether people trust the health professional who advises them, whether they trust the public institution that issues the recommendation, and whether they trust the information environment in which they encounter both [7]. All three are under strain in CEE, and they are not interchangeable, since a clinician may retain personal credibility that the ministry recommending the same vaccine has lost.

It is useful to separate the gaps that produce this pattern, because they call for different remedies. Health-system gaps concern how immunisation is financed, delivered, and recorded: the Czech Republic remains one of the last countries in Europe not to permit vaccination in community pharmacies, and immunisation registries across the region are only partly interoperable. Health-workforce gaps concern the confidence and capacity of the professionals who deliver the recommendation, and interviews with vaccine providers in four European countries, Romania among them, found hesitancy present in every country studied, with fear of side effects the most frequently reported concern [8]. Caregiver gaps concern the beliefs and circumstances of parents and patients. In Poland, refusals of mandatory childhood vaccination have risen steeply, with fear of adverse effects and the perception that children receive too many vaccines early in life among the dominant reported reasons [3]. Geographic and social gaps concern who is reached at all: coverage falls in rural areas where contact with services is thinner, and most sharply in marginalised communities, where Roma children across twelve Central and South-East European countries were found to have roughly a third of the odds of non-Roma children of being vaccinated against diphtheria–tetanus–pertussis and polio, and under two fifths of the odds for measles–mumps–rubella [9].

This decline has already been mapped. A 2025 regional meeting and its accompanying white paper set out the main barriers to confidence across CEE, among them digital misinformation, distrust of public institutions, low health literacy and low vaccine literacy, and uneven access, and called for a shift from describing the problem to acting on it [4,10]. What is already known can be stated briefly. Confidence in CEE has been below the European average for at least a decade. Its determinants are dominated by trust in institutions and in health professionals rather than by knowledge deficits. The post-communist institutional legacy is an identifiable and measurable contributor, since analysis of survey data from former communist countries shows that longer individual exposure to communist rule predicts lower reported confidence in vaccine safety and efficacy, mediated by generalised distrust of government rather than by any assessment of the vaccines themselves [6,11]. What is not known, and what motivated the 2025 meeting, is which of the available responses regional stakeholders regard as both effective and actually deliverable under existing budget, workforce, and political conditions. The 2025 meeting was convened to answer the first half of that question by mapping the barriers. The 2026 meeting reported here was convened to answer the second. The present Perspective is the second stage of that programme.

This Perspective draws together the 2026 meeting that set out to move from that diagnosis to action, distilling the convergent judgement of experts from across the region into a practical agenda for the next two years. After describing how the meeting was run, we follow the thread that connected the contributions: trust as the foundation of confidence; primary care and trusted community figures as the most feasible levers; access, equity, and system design as what convert confidence into coverage, including the work of reaching under-vaccinated groups; and structural reform, being slower but still necessary.

## 2. Meeting Design and Synthesis Approach

On 14 May 2026, an international meeting titled “Building Vaccine Confidence in Central and Eastern Europe through Multi-Stakeholder Interventions” was held at the Romanian Parliament, organised with COPAC (the Romanian Coalition of Organisations of Patients with Chronic Diseases) and the Active Citizenship Network. It was the second stage of a continuing programme. A 2025 meeting mapped the regional confidence gap, and the 2026 meeting focused on interventions feasible within a 12-to-24-month horizon. The meeting pursued three explicit objectives: to establish which barriers to vaccine confidence regional stakeholders consider most consequential in their own systems; to identify interventions judged both effective and deliverable within 12 to 24 months; and to agree an explicit division of responsibility for those interventions among professional bodies, public authorities, civil society, and industry. The meeting addressed vaccine confidence across the life course, spanning childhood, adolescent, and adult immunisation, rather than any single vaccine or age group. Around twenty-five speakers, among some fifty participants in all, contributed from Romania, Poland, the Czech Republic and Bulgaria, together with international contributors from Israel, France, the United Kingdom and Italy. They spanned public-health authorities and funders, hospital and primary care, paediatrics, obstetrics, pulmonology, epidemiology, sociology, patient-rights bodies, and patient organisations. All spoke in a professional capacity and held senior clinical, academic, regulatory, or civil-society positions in their own countries, including national immunisation-programme and public-health officials and the leadership of patient-rights and patient organisations. Country contributions were structured against three shared prompts: the most important confidence barrier today, where the system breaks from the speaker’s own vantage, and one feasible intervention in 12 to 24 months. Three instruments structured the day. The shared prompts fixed the comparison across countries; moderated panel discussion and an open floor allowed contributions to be challenged and refined in real time; and a live electronic poll put the preliminary findings of a separate expert survey back to the room for reaction. The proceedings were recorded and transcribed in full. Participants were invited experts identified by the organising partners to represent the main stakeholder groups and countries relevant to vaccine confidence in the region; the meeting was not designed as a representative sample.

The synthesis reported here followed a defined procedure. The meeting was recorded and transcribed in full, and that transcript, together with the speakers’ presentations and written contributions, formed the material for analysis. The writing group examined this material through thematic analysis, coding it inductively so that the themes emerged from the contributions themselves rather than from a framework fixed in advance. The provisional themes were then circulated to all co-authors, each of whom had taken part in the meeting and could check them against their own record of the discussion, and were revised through repeated exchange until the group reached consensus. Each theme was cross-checked against the published literature, and only themes voiced by speakers from more than one country or stakeholder group were retained. This procedure produced the four themes that organise Section 3, Section 4, Section 5 and Section 6: eroded institutional trust as the substrate on which misinformation operates; the primary-care encounter and the locally trusted community figure as the most feasible near-term levers; access, equity, and system design as the means by which restored confidence becomes recorded coverage; and structural reform, judged necessary but slower than the 12-to-24-month horizon. A fifth, cross-cutting theme concerned the division of labour among stakeholders and the conditions under which industry involvement is acceptable.

The meeting also included a short interactive session in which the preliminary findings of a separate expert survey, an anonymous and voluntary consultation of regional stakeholders on barriers, interventions, and stakeholder roles, were re-examined with participants through a live electronic poll. That exercise informally corroborated the directions emerging from the discussion. Because the survey is the basis of a separate, dedicated quantitative study, we do not report its rankings here. The present Perspective draws mainly on the qualitative content of the presentations and discussion and refers to the live poll only where it informally corroborates that content. Statements attributed below to “the meeting” reflect the convergent views expressed by its speakers, not population estimates.

## 3. Trust Is the Substrate of Confidence

The most consistent message across the presentations was that the core problem is eroded trust, and that misinformation does its damage by moving into a trust vacuum that already exists. The barriers that speakers emphasised most were digital misinformation and distrust of authorities, rather than the structural barriers of cost or distance. Participants from Romania described what they called a “perfect puzzle” specific to the region: distrust in central and local authorities, a politicised vaccination debate, low perception of disease risk, low health literacy, and misinformation, including anti-vaccine networks amplified by a minority of professionals. The live poll informally corroborated this, with trust- and information-related barriers again ranked ahead of access. Speakers described four recurring forms of the misinformation circulating in the region. The first is recycled global content, above all the discredited claim linking MMR vaccine to autism and assertions that COVID-19 vaccines impair fertility. The second is content built on partial truths, in which real safety data are stripped of their denominator or their comparator. The third, and the form participants considered most specific to the region, is sovereignty and control framing, in which vaccination is presented as an external imposition, whether by the European Union, by foreign governments, or by multinational manufacturers, on populations whose recent history gives such framings unusual traction. The fourth is the promotion of pseudomedical alternatives, which Polish contributors described as an organised commercial sector rather than a fringe belief. What gives all four unusual purchase, speakers argued, is domestic amplification: narratives carried by a familiar health professional or a political figure acquire a credibility that anonymous online content alone would never gain. Much of this content recycles claims that the scientific record has directly refuted, from the discredited 1998 study linking the MMR vaccine to autism, whose fabrication was later documented in detail [12] and whose central claim has since been contradicted by a meta-analysis of ten studies covering more than 1.25 million children [13], to unfounded assertions about COVID-19 vaccines and fertility [14], while large real-world safety studies have characterised the actual risk profile of these vaccines [15,16].

Misinformation built on partial truths, such as real data taken out of context or marginal evidence exaggerated, is the hardest to counter, because it cannot simply be dismissed as false. One example discussed at the meeting illustrates the point: claims linking COVID-19 vaccines to infertility have persisted despite a systematic review that found no such association [14]. The reputational damage of fraudulent research can persist for years, as the long aftermath of the discredited 1998 measles–mumps–rubella paper shows [12]. Social-media design compounds the problem by rewarding emotionally charged content and by sustaining closed communities that outside messages rarely reach.

Beyond the content of misinformation itself, a further explanation for the durability of vaccine hesitancy lies in how individuals weigh risk. Behavioural evidence indicates that people frequently judge the risk of a vaccine reaction as more threatening than the risk of the disease it prevents, even where the disease risk is objectively larger, a pattern consistent with omission bias, the tendency to see harm that follows from an action as worse than equivalent or greater harm that follows from inaction [17,18]. This is compounded by optimism bias, the belief that a given risk is more likely to affect other people than oneself, which underlies the common reasoning that “it will not happen to me” and has been documented specifically in parental vaccination decisions [18,19]. Read this way, part of the region’s confidence problem is not only what citizens are told about vaccines, but how the comparison between disease risk and vaccination risk is made available to them; when the disease side of that comparison stays abstract while the reaction side remains vivid and personal, a single dramatic anecdote will keep outweighing the statistics, however rare the event it describes.

Beneath these mechanics, speakers from Poland, the Czech Republic, and Romania independently identified a regional driver: a historically conditioned distrust of public institutions inherited from the communist era, when public-health initiatives were experienced as paternalistic and imposed from above. This is a legacy of the transition rather than of centralised delivery as such. Highly centralised immunisation programmes, including those operating in countries still governed as one-party states, sustain very high routine childhood coverage [1], and coverage in CEE itself stood at or above 95% in the late communist and early post-communist period [2]. What changed was not administrative capacity but the credibility of the institutions exercising it: once participation was no longer effectively compulsory and the state’s authority was openly contested, coverage became contingent on a trust that had never had to be earned. Quantitative support for this reading comes from cross-national survey analysis showing that longer individual exposure to communist rule predicts lower confidence in vaccine safety and efficacy, an association the authors attribute to reduced generalised trust in government rather than to any appraisal of the vaccines [11]. On this reading, a citizen who questions a vaccine is often questioning not the science but the credibility of the institution that recommends it. The practical implication, emphasised by colleagues from Poland, is to move away from paternalism and rebuild trust within the individual clinical relationship, while recognising that the institutions themselves still operate in a paternalistic way, which makes this as much an institutional challenge as a clinical one.

## 4. Primary Care and Trusted Community Figures: The Feasible Levers

If trust is the substrate, the meeting was equally clear about where it can most realistically be rebuilt in the near term. Two levers recurred across countries as both high in expected impact and achievable within a 12-to-24-month horizon: the primary-care encounter and the locally trusted figure. Both work through the same mechanism identified in Section 3, the credibility of the person doing the recommending, rather than on the supply of information.

The first lever is the primary-care clinician, and the recommendation that follows is to invest in continuing professional education on vaccine communication. Speakers returned repeatedly to the strength of a clear personal recommendation: the clinician who knows the patient remains the most persuasive voice, and Polish contributors framed the single most feasible intervention as a strong, confident recommendation delivered within an unhurried primary-care relationship. That confidence cannot be assumed. The Czech contribution described very low uptake even among health workers and a tendency to present vaccination as something “special” or risky, while Romanian speakers noted that a minority of professionals actively amplify anti-vaccine messages. Both sides of that judgement, the power of the recommendation and the fragility of the workforce delivering it, are well documented. A comprehensive review of the behavioural science of vaccination concluded that provider recommendation is among the most consistent correlates of uptake, and that interventions acting directly on behaviour and on the ease of vaccinating outperform those that attempt to change beliefs through information alone [20]. Evidence on how the recommendation is delivered is equally specific: in videotaped paediatric consultations, a presumptive opening that assumed vaccination would proceed was followed by substantially less parental resistance than a participatory opening that invited a decision, and providers who pursued their recommendation after initial resistance ultimately secured acceptance in about half of those encounters [21]. The reservations voiced about the workforce are equally well evidenced. Interviews with vaccine providers in four European countries, Romania among them, found hesitancy present in every country studied, with fear of side effects the most frequently reported concern and providers often uncertain how to answer patients’ questions [8]. A national survey of general practitioners in France, conducted after a period of vaccine controversy, found that a substantial minority did not consistently recommend certain vaccines, and that recommendation behaviour tracked the physician’s own trust in health authorities and comfort in explaining vaccines rather than their clinical knowledge [22]. The implication is that communication is a clinical skill to be taught and maintained, not an innate trait, and that continuing professional education, from the medical curriculum through to in-service training, is the most direct way to equip primary care to hold the conversations that change minds. Crucially, this education has to move clinicians away from the paternalistic, information-deficit posture that the region’s history has made counterproductive, and towards a dialogue that engages the patient’s actual concerns.

The second lever extends the same logic beyond the clinic to the trusted figures embedded in communities that the health system reaches least well. Where institutional credibility is weakest, speakers argued, the persuasive voice is often not the official one but the local one: the community health mediator, the religious or community leader, the genuinely trusted media voice. Israeli contributors described engaging community and religious leaders and tailoring outreach to specific subpopulations in which uptake gaps are otherwise hidden, and Romanian speakers pointed to health mediators as the working solution in marginalised communities, including Roma populations. These figures succeed precisely because they already hold the trust that public institutions have lost, and because they can tailor both the message and its language to the audience. Resourcing them, and pairing them with clinical services rather than treating them as a substitute, is a concrete and fundable step for the near term. Table 1 summarises how these and related priorities were expressed across the participating countries.

The clinician’s personal recommendation is strongest when it is reinforced, rather than undermined, by the wider information environment. Evidence from COVID-19 vaccination campaigns shows that consistent, targeted, and coordinated messaging across institutions and professional bodies is a precondition for the frontline clinician’s recommendation to be trusted, since fragmented or conflicting guidance from different authorities reopens exactly the doubts a good consultation is meant to close [23,24]. A systematic review of strategies to address vaccine hesitancy found that dialogue-based and multi-component interventions, including those that engaged and supported frontline providers, were among the most effective at increasing acceptance, which suggests that the effectiveness of “the professional voice” depends as much on how clinicians are trained and supported as on the fact that they speak [25]. The practical implication for CEE is that continuing professional education should be designed and timed together with public communication, rather than run as a parallel track, so that the same core message reaches patients through every voice they encounter.

The discussion also converged on a division of labour, which is set out here against the specific interventions, because each of them fails in a characteristic way when the wrong actor is asked to lead it. Professional bodies and medical faculties are the natural owners of continuing professional education on vaccine communication, since they control curricula, accreditation, and continuing-education credit; a training programme without their endorsement reaches few clinicians. Public authorities, meaning health ministries, public-health institutes, and insurance funds, hold the levers that no other actor can pull: reimbursement, the legal basis for pharmacy administration, the immunisation registry on which reminder and recall depends, and the consistency of national communication. Civil-society and patient organisations hold what institutions have lost in parts of the region, which is standing within under-vaccinated communities, and they are the appropriate leads for outreach through health mediators and locally trusted figures, provided they are funded as continuing partners rather than engaged campaign by campaign. Primary-care clinicians and, for adults, pharmacists are the point of delivery for all of it, which is why measures that close missed opportunities depend less on new resources than on the permissions and protocols these professionals work under. Industry was seen as a partner that contributes resources and evidence, not as the lead on public-facing confidence work, a boundary that matters for how partnerships and their disclosures are framed, including in this Perspective.

This allocation is consistent with the roles already set out in European policy. The European Immunization Agenda 2030 assigns complementary responsibilities to Member States, health professionals, civil society, and national immunisation technical advisory groups [26], and the Council Recommendation on strengthened cooperation against vaccine-preventable diseases makes Member States responsible for national schedules and for health-worker training while establishing the Coalition for Vaccination as the vehicle through which professional bodies act [27]. What the meeting added to those frameworks was regionally specific. Where institutional trust is weak, the credibility of whoever delivers a message determines whether it is heard at all, so the division of labour is not merely administrative tidiness but part of the intervention: the same recommendation carries differently depending on who makes it. This is also why the participants were unwilling to see confidence-building led by any actor whose motives could be questioned, and why they placed outreach with organisations that communities already trust. A message can be both evidence-based and locally adapted without compromise, since tailoring changes how content is delivered rather than the underlying evidence. A five-country survey found that appeals combining a clear health-outcome message with a trusted messenger, particularly a healthcare provider, outperformed generic appeals, confirming that message content mattered less than who delivered it and how it was framed for the audience [28]. Narrative and storytelling formats have shown similar value for audiences with lower health literacy, working as an accessible vehicle for the same clinical evidence rather than a simplification of it [29], and trust in health professionals, health literacy, and self-efficacy have each been shown to independently predict whether a hesitant audience acts on a message, offering concrete dimensions along which CEE campaigns could segment their communication [30]. For the region’s professional-education and community-figure strategies, this argues for a shared evidence base rather than a shared script: one set of facts, translated into a technical framing for clinical audiences, a narrative framing for lower-literacy or historically distrustful groups, and a peer framing for younger audiences.

## 5. Turning Confidence into Coverage: Access, Equity, and System Design

Alongside the trust narrative, the meeting produced a concrete argument about system design, together with regional evidence that it works. This section concerns the point at which a person who is willing to be vaccinated either becomes a recorded dose or is lost. A frequently cited example came from Romania’s national health insurance fund, where the introduction of direct reimbursement and administration in pharmacies was followed by large year-on-year increases in uptake across several vaccines [31]. This is consistent with the international evidence, since a systematic review and meta-analysis of thirty-six studies found that involving pharmacists in immunisation, whether as educators, facilitators, or administrators, increased vaccination coverage in every study reviewed [32]. Poland described a similar agenda, including reimbursement of RSV and HPV vaccination, pharmacy administration, and a simplified patient pathway. The Czech Republic illustrated the opposite case, as one of the last countries in Europe without pharmacy vaccination and with low uptake even among health workers. Digital tools support these strategies when used well: interoperable immunisation registries that let any clinical contact become a vaccination opportunity, real-time monitoring of online misinformation, and clinicians who reach the public directly through trusted digital channels. Registries also enable the least contentious intervention available: a Cochrane review of fifty-five studies found that patient reminder and recall increased immunisation uptake by about eight percentage points (risk ratio 1.28, 95% confidence interval 1.23 to 1.35), with text-message reminders the most effective single method [33].

The mirror image of access is the missed opportunity, meaning a clinical engagement in which patients with indications for receiving vaccines cannot get vaccinated during the same engagement due to system barriers. Pulmonology clinics see high-risk adults with chronic respiratory diseases yet often cannot give influenza or pneumococcal vaccines or reach the immunisation registry because of system barriers, resource constraints and cost, a gap that European respiratory-health advocacy has argued should be closed as a matter of policy [34]. Obstetric care reaches pregnant women but frequently fails to deliver maternal vaccination reliably because of system barriers, gaps in physicians’ knowledge, and unsettled assumptions about which physician is responsible for promoting vaccination. Closing these gaps, by vaccinating at discharge after a pulmonary exacerbation or by building maternal vaccination into antenatal care, calls for small and specific changes to protocols and permissions rather than new campaigns, and is among the least costly ways to raise uptake.

Boosting coverage also means reaching the groups that current programmes miss, which is where inequities are widest. Speakers described how under-vaccination concentrates in particular populations and places: marginalised communities, including Roma, where stigma, discrimination, and practical obstacles such as missing identity documents or no registered family doctor depress uptake, and rural areas where contact with services is thinner. The first of these gaps has been quantified across twelve Central and South-East European countries, where Roma children had roughly a third of the odds of non-Roma children of being vaccinated against diphtheria–tetanus–pertussis and polio, and under two fifths of the odds for measles–mumps–rubella [9]. The strategies reported to work here are community-based, namely, health mediators and local figures who are trusted within these communities, flexible or walk-in vaccination centres, and targeted catch-up campaigns delivered with non-governmental and community partners. Because national averages can hide these gaps, monitoring and acting at the level of specific neighbourhoods and groups is itself part of an effective coverage strategy.

Respiratory syncytial virus (RSV) prevention offers a template that can be transferred. The recent French rollout of maternal vaccination and the infant monoclonal antibody nirsevimab succeeded because it was planned and coordinated from end to end, with open parental choice, professional cohesion, supportive media, and weekly surveillance. Real-world studies have since confirmed a substantial reduction in infant hospitalisations [35], and uptake exceeded expectations to the point of product shortage. The wider lesson is not specific to RSV: anticipate demand, make the safe option easy, publicise effectiveness and impact, and sustain public demand for prevention from one season to the next.

Table 2 makes the trade-off explicit. The measures with the strongest evidence base and the shortest time to effect are those that reduce friction at the point of vaccination, rather than those that attempt to change beliefs directly. The measures with the largest potential impact are those that act on the substrate of trust, and these are precisely the ones that fall outside the meeting’s 12-to-24-month horizon. The practical implication for the region is to fund the near-term levers while beginning the structural work, rather than treating the two as alternatives. Table 1 shows how each lever maps onto the specific barriers and constraints of the participating countries, which is where decisions about sequencing and local adaptation have to be made.

**Table 2:** Comparison of the interventions prioritised at the 14 May 2026 meeting, by mechanism, implementation difficulty, expected impact, and evidence base. Difficulty and impact ratings are the authors’ synthesis of the meeting discussion read against the cited literature. They are analytic judgements offered to support prioritisation, not measured quantities, and no formal cost-effectiveness analysis was undertaken. The final column grades the certainty of the evidence supporting each intervention as high, moderate, low, or very low. These ratings were made by the authors using the GRADE domains of study design, consistency, precision, and directness, and they take as their starting point the certainty ratings reported by the source reviews themselves. They are a pragmatic appraisal rather than a formal GRADE exercise, which would require a systematic review of each intervention. Ratings are downgraded for indirectness wherever the underlying evidence comes from health systems unlike those of Central and Eastern Europe, or from age groups other than the one at issue. The final row is included for contrast, since the structural agenda of Section 6 falls outside the 12-to-24-month horizon that defines the others.

Intervention	Mechanism	Implementation Difficulty and Main Requirement	Expected Impact and Time to Effect	Evidence Base	Certainty of the Evidence
Continuing professional education on vaccine communication for primary care	Strengthens the clinician’s recommendation, the most consistent single correlate of uptake	Low to moderate. Uses existing continuing-education structures; the cost is faculty time and protected training hours. No legislation required	Moderate to high; begins within one training cycle (6–12 months) but is constrained by consultation time	Provider recommendation associated with uptake [20]; presumptive delivery reduces resistance [21]; provider hesitancy documented in Europe including Romania [8,22]	Low. Face-to-face education of parents may improve early childhood vaccination status (RR 1.20, 95% CI 1.04–1.37), but is downgraded for risk of bias and serious inconsistency [36]. The two randomised trials that tested clinician communication training itself were null, including one measuring immunisation status at 19 months of age [37]
Resourcing locally trusted community figures and health mediators	Substitutes credible local messengers where institutional trust is lowest and reaches groups outside routine service contact	Moderate. Low unit cost, but requires stable funding, formal recognition and linkage to clinical services rather than project-by-project financing	High within targeted communities, limited effect on the national average; 12–24 months	Roma children markedly under-vaccinated across twelve CEE and South-East European countries, with access the principal barrier [9]	Low. Lay health workers and community-directed education raise childhood immunisation uptake (RR 1.22, 95% CI 1.10–1.37) [38], and community leader involvement combined with a provider intervention is of moderate certainty in low- and middle-income settings [39]. Downgraded for indirectness: no controlled evaluation of health mediators has been published in Central and Eastern Europe
Pharmacy-based vaccination with direct reimbursement	Removes appointment, distance, and cost friction, converting existing willingness into recorded doses	Moderate to high. Requires regulatory or legislative change, pharmacist training, and registry access. Already in place in Romania and Poland, absent in the Czech Republic	High and rapid where enacted; measurable within one season	Systematic review and meta-analysis of 36 studies: pharmacist involvement increased coverage in every study reviewed [32]; Romanian reimbursement and pharmacy administration followed by large year-on-year increases [31]	Moderate for adults, in whom pharmacist involvement increases uptake (pooled RR 1.14, 95% CI 1.12–1.15) [32]. Not applicable to children aged 12 to 24 months, who cannot be vaccinated in a pharmacy in Romania or Poland, where pharmacist administration is restricted to adults
Closing in-clinic missed opportunities (antenatal care, discharge, pulmonology)	Uses clinical contacts that already occur, requiring no new demand generation	Low. Protocol and permission changes plus registry access; no new infrastructure and no campaign	Moderate, concentrated in high-risk groups; within 12 months	Advocated as a policy priority for respiratory patients [34]; consistent with evidence that reducing friction outperforms information provision [20]	Low. The only systematic review specific to missed opportunities rests on six studies, all from the United States and all at high risk of bias [40]. A cluster-randomised trial of presumptive delivery, with immunisation status at 19 months as its primary outcome, was null [37]
Reminder and recall linked to immunisation registries	Acts on forgetting and scheduling rather than on belief	Moderate, and contingent on registry interoperability, which is itself a structural constraint	Moderate and predictable; within 12 months once registries permit it	Cochrane review of 55 studies: risk ratio 1.28 (95% CI 1.23–1.35), about 8 percentage points absolute; text messages the most effective single method [33]	High for childhood immunisation as a whole: the Cochrane childhood subgroup gives RR 1.22 (95% CI 1.15–1.29) across 23 studies, with high certainty for postcard, text-message, and auto dialer reminders [33]. Moderate for this region, since no trial has been conducted in Central or Eastern Europe and delivery depends on registry completeness
Consistent, coordinated public communication and infodemic monitoring	Removes the contradictory institutional signals that undermine the clinical encounter	Politically demanding but financially modest; requires inter-institutional discipline rather than budget	Uncertain in magnitude, but a precondition for the other levers; continuous	Coordinated messaging identified as a precondition for a trusted clinician recommendation [23,24]; national information campaign associated with recovery of HPV uptake in Denmark [41]	Very low. Public information campaigns used alone have not been shown to raise childhood coverage, and no randomised trial measures coverage in the second year of life; the supporting evidence is for face-to-face and community-directed education rather than mass communication [39]
Structural reform: transparency, interoperable data, life-course policy (shown for contrast)	Rebuilds institutional credibility and enables every other lever	High. Requires legislation, inter-ministerial agreement and sustained funding	Potentially the largest, but beyond the 12–24-month horizon	French mandate extension shows structural change plus communication can raise coverage, though transferability to low-trust settings is uncertain [42]	Not gradable. These are governance instruments rather than discrete interventions, and no controlled study compares immunisation programmes with and without them

Grading the evidence in this way exposes two qualifications that matter more than any single rating. The first concerns age. The interventions above span the life course, and their evidence bases do not align neatly with a single age band. For children in the second year of life, the most directly relevant syntheses are the Cochrane review of interventions to improve childhood immunisation coverage [39] and the Cochrane review of patient reminder and recall [33], the latter providing the only high-certainty estimate in the table. Even there, the evidence is not perfectly aligned: the headline estimates in the childhood immunisation review are for a third dose of diphtheria–tetanus–pertussis-containing vaccine by one year of age, and only its secondary outcome, full vaccination by two years, falls squarely in the 12-to-24-month window. Pharmacy-based vaccination sits at the opposite extreme, since it cannot lawfully be delivered to this age group in Romania or Poland and belongs to the adult and adolescent part of the agenda.

The second qualification concerns setting, and it is the more consequential of the two. Much of the childhood evidence, including the Cochrane review cited above, was generated in low- and middle-income countries, where the binding constraint on coverage is physical access to services [39]. In most of Central and Eastern Europe, access is close to universal and the binding constraint is confidence, so interventions that work by bringing services closer to families may not transfer, whatever their measured effect elsewhere. We have therefore downgraded certainty for indirectness wherever the evidence derives from such settings. It should be stated plainly that no controlled evaluation of any of these interventions has been published from Central or Eastern Europe itself. That absence is a finding in its own right, and it is the strongest argument for the evaluation agenda proposed in Section 8: the region is currently importing effect estimates it has never tested at home.

## 6. Structural Reform: Desired but Stalled

A recurring tension at the meeting was the distance between what experts believe matters and what they believe can be done quickly. Items rated high in impact but low in feasibility, such as institutional transparency on vaccine decisions and safety data, interoperable immunisation data, and an explicit life-course immunisation policy, were seen as the political work of the next several years rather than near-term gains. The corollary, stated firmly, was that sporadic, campaign-style efforts are not enough, and that consistency and systemic change are required.

Experience from outside the region indicates both what structural reform can achieve and why it cannot simply be transplanted. France extended mandatory infant vaccination from three diseases to eleven in 2018, a measure followed by increased coverage not only for the newly mandated vaccines but also for vaccines outside the mandate, which the authors attributed in part to the accompanying communication strategy rather than to compulsion alone [42]. Denmark offers the closer analogy for a confidence crisis: HPV vaccination uptake collapsed after sustained negative media coverage and then recovered substantially during a national information campaign, indicating that lost confidence can be regained, but that regaining it requires deliberate, funded, and continuous communication by public authorities rather than a single campaign [41]. Two cautions follow for CEE. First, mandates presuppose the institutional legitimacy that is precisely what is contested across much of this region, and in low-trust settings, compulsion risks confirming the narrative of imposition described in Section 3. The Polish contribution, notably, framed legal instruments as a tool against organised anti-evidence practice rather than against hesitant parents. Second, the Danish recovery rested on a health system whose data infrastructure allowed the decline to be detected quickly, which is not yet the case across much of CEE. The transferable elements are therefore the coordinated communication and the surveillance capacity that make such a response possible, rather than the mandate itself.

Two framings can make this structural agenda more achievable politically. The first is economic. Vaccination is a high-return, life-course investment for ageing societies, in which healthy older adults are an asset rather than a cost, and failure to vaccinate drives clinical, productivity, and care costs. Assessing immunisation for its value, and not only its budget line, helps make that case to funders and treasuries, a framing that decision-makers increasingly accept [4]. The second is European. The emerging policy dialogue around an EU Respiratory Health Plan and a Council Recommendation on immunisation against respiratory infections offers a frame within which national reforms can be situated, provided that civil-society and patient organisations, currently under-used, are treated as strategic communication partners rather than an afterthought [34].

## 7. Strengths and Limitations

This work is a meeting-informed Perspective, not a representative population study. Participants were selected stakeholders invited to represent key groups and countries, so country and sector representation was limited and the synthesis may be affected by selection and interpretation bias. The evidence base is the presentations, moderated discussion, and open-floor contributions of that meeting, cross-checked against the published literature. It is not a survey, a systematic review, or an original quantitative analysis, and the statements attributed to the meeting are convergent expert judgements rather than population estimates. The meeting received partial sponsorship from Pfizer; to protect independence, the sponsor had no role in the design of the meeting, the content of the speakers’ contributions, the analysis or interpretation, the writing of this manuscript, or the decision to submit it, and the views and priorities reported here are those of the participants and authors. The strengths of the work are its cross-country, cross-role breadth, its grounding in frontline experience, and its focus on interventions judged feasible within a short horizon.

A second class of limitation concerns implementation rather than evidence. The recommendations set out here would have to be delivered across health systems that differ in ways that materially affect their feasibility. Reimbursement and procurement decisions are national, so a lever that is already funded in one country, such as pharmacy administration in Romania and Poland, requires primary legislation in another, such as the Czech Republic. Reaching marginalised populations, and Roma communities in particular, is constrained not only by trust but by administrative preconditions such as identity documentation and registration with a family doctor, which lie outside the health ministry’s control [9]. Digital misinformation is not amenable to a 12-to-24-month fix at all, since platform design and cross-border information flows are governed at the European rather than national level and monitoring capacity across most of the region is nascent. Administrative fragmentation between ministries of health, insurance funds, public-health institutes, and local authorities means that several of the proposed measures have no single accountable owner. Workforce capacity is a further binding constraint, because the primary-care lever assumes consultation time and staffing that are already stretched. Finally, immunisation information systems in much of the region are not interoperable, which limits both targeted outreach and any subsequent evaluation of whether these measures worked.

A third limitation concerns what this article can and cannot establish. The interventions prioritised here were judged feasible and effective by the participants. Where published evidence exists we have cited it, but its strength varies considerably across the measures compared in Table 2, and most of it derives from settings with higher baseline institutional trust than the countries discussed here, so transferability should be treated as a hypothesis rather than an assumption. We also do not report the quantitative rankings from the accompanying expert survey, which are the subject of a separate study. The live poll is referred to only where it informally corroborated the qualitative discussion, and no statistical inference should be drawn from it.

## 8. Conclusions: A 12–24-Month Agenda for Central and Eastern Europe

**Figure 1:**
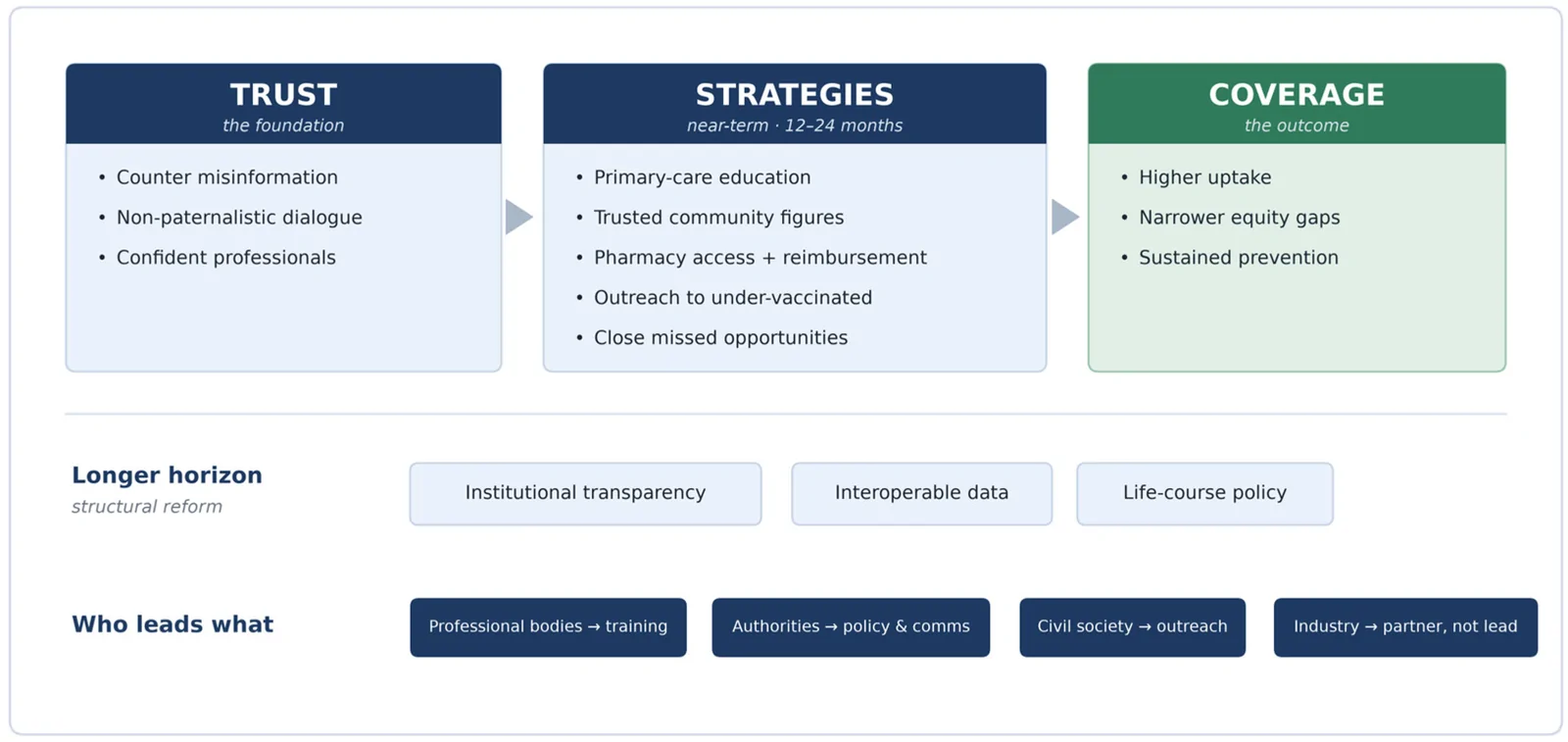
A practical agenda for boosting vaccine coverage in Central and Eastern Europe, as synthesised from the 14 May 2026 meeting. The figure is read from the bottom upward as a three-tier chain. The foundation tier is trust: where it is absent, the tiers above it do not transmit, which is why identical interventions produce different results in different countries. The near-term tier collects the strategies judged deliverable within 12 to 24 months, namely, continuing professional education on vaccine communication for primary care, resourcing locally trusted community figures, extending pharmacy-based vaccination with direct reimbursement, closing missed opportunities in clinical settings, and targeted outreach to under-vaccinated and marginalised groups; these measures are compared on feasibility, resource requirement, and evidence base in Table 2. The structural tier collects reforms judged necessary but slower than that horizon, namely, institutional transparency on vaccine decisions and safety data, interoperable immunisation data, and an explicit life-course immunisation policy, each of which requires legislation or inter-ministerial agreement rather than programme funding. The division-of-labour band underpinning all three tiers assigns clinical training to professional bodies; policy, reimbursement, and public communication to public authorities; outreach to vulnerable populations to civil-society and patient organisations; and a contributing rather than leading role to industry. That allocation is what protects the credibility of the agenda as a whole. The chevron arrows show the direction of the chain, from trust through the near-term strategies to coverage, and the arrows in the lower band link each stakeholder group to the task it leads. Blue denotes the inputs and green the outcome; colour carries no quantitative meaning.

The meeting points to an agenda that is both feasible and specific (Figure 1). In the near term, over 12 to 24 months, the priorities are to invest in continuing professional education for primary care on vaccine communication; to resource locally trusted community figures and tailor messages to particular audiences; to reduce friction by extending pharmacy-based vaccination and direct reimbursement and by closing in-clinic missed opportunities; to reach under-vaccinated and marginalised groups through community-based outreach so that gains in coverage also narrow inequities; and to protect the credibility of the whole effort through a clear division of labour, in which professional bodies lead training, public authorities lead policy and communication, civil society leads outreach, and industry partners rather than leads.

In parallel, the structural agenda of institutional transparency, interoperable data, and an explicit life-course immunisation policy should be advanced steadily, anchored in the economic case and in the European policy dialogue. This meeting-informed Perspective indicates that improving vaccine uptake in Central and Eastern Europe requires more than additional information campaigns. Stakeholder contributions highlighted interlinked barriers of weakened trust, inconsistent communication, missed vaccination opportunities, and inequitable access. Near-term priorities include strengthening vaccine communication in primary care, engaging locally trusted community figures, reducing practical barriers through reimbursement and, where appropriate, pharmacy-based vaccination, closing missed opportunities in clinical settings, and reaching under-vaccinated communities through community-based outreach. These proposals should be interpreted as a stakeholder-derived action agenda rather than as population-level evidence of intervention effectiveness. Subsequent studies should evaluate these strategies using transparent, country-specific implementation indicators and measurable vaccine uptake outcomes.

## Figures and Tables

**Table 1:** Country priorities expressed at the 14 May 2026 Bucharest meeting (Romania, Poland, the Czech Republic, and Bulgaria, with Israel included only as an international comparator, from outside the CEE region). The table reflects the speakers’ stated views and is a qualitative synthesis, not a representative survey. Each entry summarises the contributions of one or more speakers from that country, not a single individual’s position or a population estimate. Context indicators in the second column are drawn from published sources and situate the stated views against measured coverage and confidence. They were not presented at the meeting. The final column combines constraints raised in discussion with those identified in the cited literature. Evidence on the effectiveness of the listed interventions is summarised in Table 2.

Country	Context Indicators (Published Data)	Most Emphasised Barrier(s)	Feasible 12–24-Month Intervention(s)	Key Implementation Challenges
Romania	MCV1 83.4% and MCV2 71.4% in 2022, down from 96 to 97% in 2005–2008; MCV2 about 62% in 2023, the lowest in the EU/EEA; 7243 measles cases and 8 deaths, January 2023–March 2024 [2]	Distrust in authorities; politicised vaccination debate; low disease-risk perception; misinformation	Pharmacy administration with direct reimbursement; in-clinic vaccination (at discharge and in antenatal care); national audit of good practice; community health mediators	Reaching undocumented and marginalised populations; wide county-level heterogeneity in coverage; politicisation of the debate limits cross-party policy continuity
Poland	Recorded refusals of mandatory childhood vaccination rose from 3437 (2010) to 48,600 (2019) and 87,300 (2023); fear of adverse effects and perceived vaccine overload dominate parental reasons [3]	Post-communist institutional distrust; misinformation and pseudomedicine	Strong personal recommendation in primary care; simplified one-visit pathway; pharmacy vaccination; legal instruments against dangerous anti-evidence practice	Organised pseudomedical sector; legal instruments against anti-evidence practice are contested; consultation time is the binding constraint on the primary-care recommendation
Czech Republic	Childhood coverage historically among the highest in the region; one of the last EU countries not permitting vaccination in community pharmacies; very low uptake among health workers (reported at the meeting)	Vaccination framed as “special” or risky; benefits under-communicated; very low health-worker uptake	Open pharmacies to vaccination; insurer data plus GP motivation; education from school to medical curricula; genuinely trusted media voices	Legislative change required to open pharmacies to vaccination; low health-worker uptake undermines the professional voice; insurer data not routinely used for outreach
Bulgaria	Among the EU Member States with the lowest agreement that vaccines are safe and important in the 2019 Special Eurobarometer [5]; regional confidence persistently below the western European average [6]	“Informed ignorance”; weak teamwork among stakeholders; misinformation	Consistency rather than campaign-style efforts; structured infodemic response; accessible information in the patient’s own language; a shift from vaccine introduction to a policy of trust, access, and accountability	Fragmented coordination among stakeholders; campaign-style rather than continuous funding; information not consistently available in patients’ own languages
Israel	International comparator (outside the CEE region); high routine childhood coverage overall, with uptake gaps concentrated in specific subpopulations (reported at the meeting)	Misinformation built on partial truths, which even informed groups and professionals can accept; uptake gaps hidden within specific subpopulations	Reliable information on both benefits and limitations of vaccines; engagement of community and religious leaders; subpopulation-targeted outreach; adult vaccination embedded in medical education	Transferability limited by a different insurance and digital-registry context; subpopulation targeting needs granular data most CEE systems do not hold

## Data Availability

The anonymised meeting synthesis supporting the findings of this article is available from the corresponding author on reasonable request.

## Abbreviations

CEE	Central and Eastern Europe
CI	confidence interval
CNAS	Romanian National Health Insurance House
COPAC	Coalition of Organisations of Patients with Chronic Diseases, Romania
COVID-19	coronavirus disease 2019
DTP3	third dose of diphtheria–tetanus–pertussis-containing vaccine
EEA	European Economic Area
EU	European Union
GP	general practitioner
GRADE	Grading of Recommendations, Assessment, Development and Evaluation
Hib	*Haemophilus influenzae* type b
HPV	human papillomavirus
MCV1/MCV2	first/second dose of measles-containing vaccine
MMR	measles–mumps–rubella
Pol3	third dose of poliovirus vaccine
RR	risk ratio
RSV	respiratory syncytial virus
UNICEF	United Nations Children’s Fund
WHO	World Health Organization
WUENIC	WHO/UNICEF Estimates of National Immunization Coverage
